# Gene expression patterns following unilateral traumatic brain injury reveals a local pro-inflammatory and remote anti-inflammatory response

**DOI:** 10.1186/1471-2164-14-282

**Published:** 2013-04-25

**Authors:** Todd E White, Gregory D Ford, Monique C Surles-Zeigler, Alicia S Gates, Michelle C LaPlaca, Byron D Ford

**Affiliations:** 1 Department of Neurobiology, Neuroscience Institute, Morehouse School of Medicine, 720 Westview Drive SW, Atlanta, GA 30310, USA; 2 Department of Biology, Morehouse College, 830 Westview Drive SW, Atlanta, GA, 30314, USA; 3 Department of Biomedical Engineering, Georgia Institute of Technology, 313 Ferst Drive, Atlanta, GA, 30332, USA

**Keywords:** Bioinformatics, Cytokine, Gene interaction hierarchy, Inflammation, Microarray, Rat, Traumatic brain injury

## Abstract

**Background:**

Traumatic brain injury (TBI) results in irreversible damage at the site of impact and initiates cellular and molecular processes that lead to secondary neural injury in the surrounding tissue. We used microarray analysis to determine which genes, pathways and networks were significantly altered using a rat model of TBI. Adult rats received a unilateral controlled cortical impact (CCI) and were sacrificed 24 h post-injury. The ipsilateral hemi-brain tissue at the site of the injury, the corresponding contralateral hemi-brain tissue, and naïve (control) brain tissue were used for microarray analysis. Ingenuity Pathway Analysis (IPA) software was used to identify molecular pathways and networks that were associated with the altered gene expression in brain tissues following TBI.

**Results:**

Inspection of the top fifteen biological functions in IPA associated with TBI in the ipsilateral tissues revealed that all had an inflammatory component. IPA analysis also indicated that inflammatory genes were altered on the contralateral side, but many of the genes were inversely expressed compared to the ipsilateral side. The contralateral gene expression pattern suggests a remote anti-inflammatory molecular response. We created a network of the inversely expressed common (i.e., same gene changed on both sides of the brain) inflammatory response (IR) genes and those IR genes included in pathways and networks identified by IPA that changed on only one side. We ranked the genes by the number of direct connections each had in the network, creating a gene interaction hierarchy (GIH). Two well characterized signaling pathways, toll-like receptor/NF-kappaB signaling and JAK/STAT signaling, were prominent in our GIH.

**Conclusions:**

Bioinformatic analysis of microarray data following TBI identified key molecular pathways and networks associated with neural injury following TBI. The GIH created here provides a starting point for investigating therapeutic targets in a ranked order that is somewhat different than what has been presented previously. In addition to being a vehicle for identifying potential targets for post-TBI therapeutic strategies, our findings can also provide a context for evaluating the potential of therapeutic agents currently in development.

## Background

It is estimated that 3.17-3.32 million persons in the United States are living with long-term or lifelong effects of traumatic brain injury (TBI) [1]. Approximately 1.7 million new TBIs are sustained each year [2] resulting in 53,000 deaths [3] and as many as 125,000 additional people that have long-term behavioral deficits [4]. Efforts to implement preventative measures for the leading causes of TBI (motor vehicle-traffic accidents, falls, and assaults) are critical [2,3] but will not eliminate TBI as a major public health problem. Development of effective clinical treatment protocols post-TBI and potential prophylactic agents for military usage are necessary to address those TBIs that cannot be prevented.

Injury to the brain results in irreversible damage at the site of impact and initiates cellular and molecular processes that lead to delayed or secondary neural injury in the surrounding tissue [5,6]. Neuroprotective strategies target these processes in an attempt to halt the progression of or prevent the delayed injury [7]. These processes include inflammation, damage to the blood brain barrier, release of excitatory amino acids, oxidative stress, cerebral edema, reduced cerebral blood flow, hypoxia, and ischemia [5,6]. In the current study, we used gene microarray analysis to determine which genes, pathways and networks were significantly altered after unilateral controlled cortical impact (CCI), an experimental model of TBI.

Microarray technology is a very powerful tool that allows the user to examine expression profiles for thousands of genes at one time. The true power of microarray technology can be maximized when the expression profiles can be attributed to significant alterations in biological functions and molecular pathways. Fortunately, the recent development of advanced bioinformatic analysis tools has made the utilization of microarray data more practical and allows for easier replication. One such tool is the Ingenuity Pathway Analysis (IPA) software program which uses a database built from published scientific literature to draw direct and indirect interactions between genes and to assign genes to specific biological functions and canonical pathways [8]. IPA was used here for functional, canonical pathway, and network analysis of the genes that were altered by TBI. We examined gene expression on both sides of the brain in order to understand the alterations both locally (ipsilateral) and remotely on the opposite side of the brain (contralateral). We observed that the contralateral side of the brain, which has often been used as a control in similar experiments, exhibited significant alterations in gene expression following TBI.

The bioinformatic analysis tools we used also allowed for both identification of key molecules and elucidation of their interactions with each other. We used these interactions to identify molecules and molecular pathways central in the response to TBI that could provide novel targets for therapeutic strategies. Understanding how local and remote gene expression profiles change following TBI, when compared to non-injured brain, will provide valuable insight into delayed neuronal injury mechanisms as well as intrinsic neuroprotective processes.

## Results

### Principal component analysis

**Figure 1:**
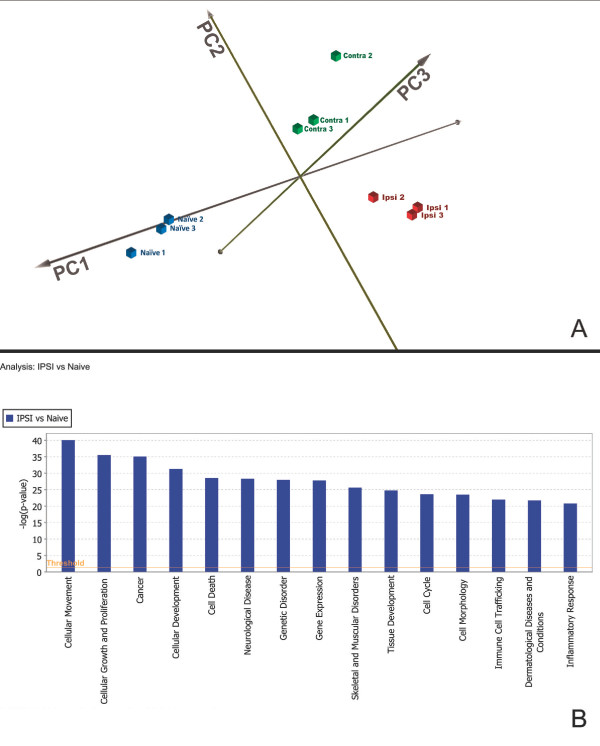
**Principal component and functional analyses. **(**A**) PCA was applied to all microarray datasets and the resulting scores for the first 3 principal components were plotted. The first 3 principal components explain 91.267% of the variance in the data. This analysis revealed clustering of datasets by injury status: Ipsilateral (Ipsi), Contralateral (Contra), and Naïve. (**B**) Analysis of the top 15 biological functions determined by IPA for the TBI-I (Ipsilateral vs Naïve) dataset demonstrates that the inflammatory response is one of the primary functions of the genes expressed after TBI. Additionally, all of the functions IPA found to be more significant than inflammatory response also have an inflammatory component (see text).

The ipsilateral, contralateral, and naïve gene datasets generated 24 hours post-TBI were analyzed by principal component analysis (PCA) to assess the variability of our microarray data. Nine principal components were generated and the first 3 principal components explain 91.267% of the variance in our microarray data. The 3D score plot generated from these identified injury status as the major source of variability (Figure 1A). Similarity between data points in the PCA was determined by the distance between the points, with shorter distance indicating increased similarity. Each data point represents one animal's gene expression profile. Ipsilateral, contralateral and naïve all clustered together by injury status and each group was well isolated from the other two groups.

### Functional analysis

A total of 69 biological functions met IPA's threshold and cutoff conditions for the TBI-I (ipsilateral vs naïve) dataset. Inspection of the top 15 biological functions associated with the dataset in IPA showed that 7 of these functions are explicitly cellular in nature; cellular movement, cellular growth and proliferation, cellular development, cell death, cell cycle, cell morphology, and immune cell trafficking (Figure 1B). Of the other top functions, 5 are disease and disorder related; cancer, neurological disease, genetic disorder, skeletal and muscular disorders, and dermatological diseases and conditions. We posited that the majority of these functional categories had an inflammatory component and inflammatory response (IR) itself was ranked as the 15th most significant biological function for our dataset. To further investigate this, we calculated the percentage of gene overlap each function had with inflammatory response. This overlap ranged from 26% to 93% with 9 biological functions falling in the 35% to 55% range (Table 1). The cumulative overlap of IR with the other 14 top biological functions was 39.3%. Therefore, we chose to focus our subsequent analysis techniques on the inflammatory response genes in this study because of the significant involvement of inflammatory genes in all of the top ranked biological functions and the role of the IR in secondary neural injury [5,6,9,10].

**Table 1:** Inflammatory nature of top biological functions

**Biological function**	**Unique genes for function**	**# of overlapping IR genes**	**% overlap**
Cellular movement	381	209	55%
Cellular growth and proliferation	583	227	39%
Cancer	663	225	34%
Cellular development	499	208	42%
Cell death	472	202	43%
Neurological disease	663	185	28%
Genetic disorder	1023	267	26%
Gene expression	441	151	34%
Skeletal and muscular disorders	538	191	36%
Tissue development	479	203	42%
Cell cycle	296	111	38%
Cell morphology	267	133	50%
Immune cell trafficking	231	216	94%
Dermatological diseases & conditions	268	147	55%

### Histology and immunohistochemistry

**Figure 2:**
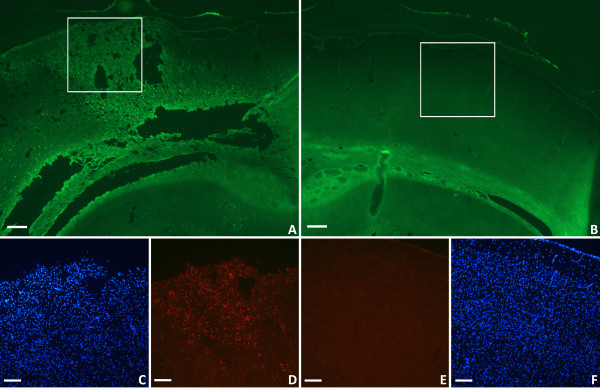
**Cortical histology following TBI. **Damage to the ipsilateral cortex is demonstrated by FJB staining (**A**). FJB staining is absent in the contralateral cortex (**B**), consistent with the lack of trauma. The damaged cortex (**C**) contains many macrophages and activated microglia as demonstrated by ED-1 immunostaining (**D**). There were no activated macrophages in the contralateral cortex (**E**) and the brain anatomy appears intact (**F**). *FJB: green; DAPI: blue; ED-1: red; Scale bars: 250 μm (***A**,**B**), 125 μm (**C**-**F**).

Histology and immunostaining were used to assess damage to and the inflammatory state of the brain. Figure 2 demonstrates cortical damage to the brain tissue ipsilateral to the injury by Fluoro-Jade B (FJB) and DAPI staining (Figure 2A,C). Near the site of impact, the integrity of the cortex is disrupted and there is obvious tissue loss. ED-1 immunostaining showed that many activated microglia/macrophages were present in the area of tissue damage (Figure 2D). The contralateral side of the brain showed no overt structural damage or ED-1 immunoreactivity (Figure 2B,E,F).

**Figure 3:**
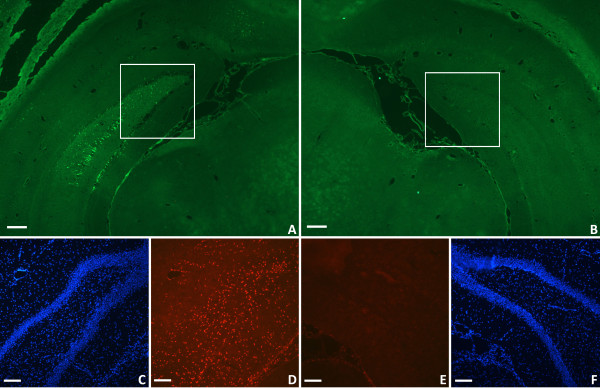
**Hippocampal region histology following TBI. **Damage to the ipsilateral hippocampal region was demonstrated by FJB staining (**A**). FJB staining was absent in the contralateral hippocampal region (**B**). While the integrity of the hippocampus appears intact ipsilaterally (**C**), many activated microglia and macrophages were present as demonstrated by ED-1 immunostaining (**D**). There were no activated macrophages in the contralateral hippocampal region (**E**) and the anatomical structure appears intact (**F**). *FJB: green; DAPI: blue; ED-1: red. Scale bars: 250 μm (***A**,**B**), *125 μm (***C**-**F**).

The hippocampal region did not display overt structural damage on either side of the brain (Figure 3C,F) but did show cellular damage ipsilaterally when stained with FJB (Figure 3A). ED-1 staining similarly showed only macrophage/microglial activation in the hippocampus on the ipsilateral side of the brain (Figure 3D). No cortical or hippocampal labeling was seen in negative control sections (data not shown). Any apparent staining in the contralateral hemisphere represented autofluorescence and was similar to levels detected in negative controls.

**Figure 4:**
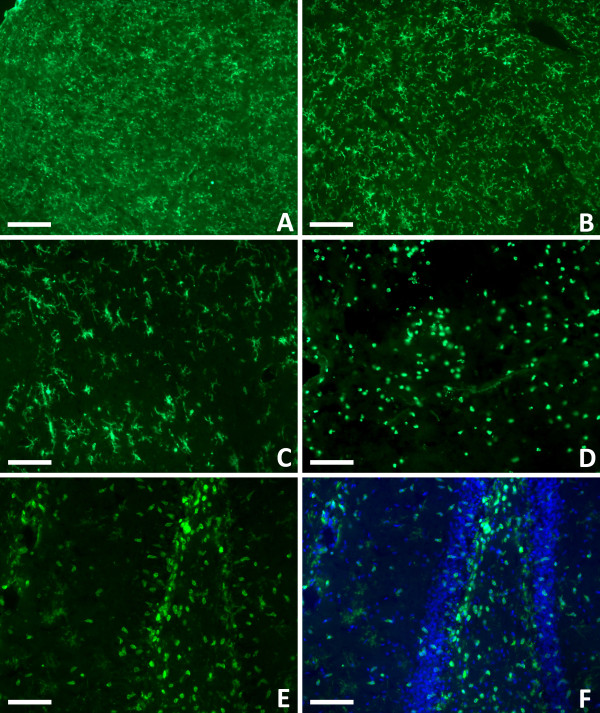
**Microglial activation in the injured brain. **CD11b immunostaining demonstrated the ramified resting morphology of microglia on the uninjured, contralateral side of the brain (**A** &**B**). This same morphology was seen on the ipsilateral side of the brain in areas less affected by the trauma (**C**). In damaged brain regions, the microglia were activated and underwent a morphological change, becoming amoeboid in shape (**D**). Both morphologies can be seen in the hippocampal region where there are amoeboid microglia in the areas of damage and ramified microglia in the surround (**E**). The same section was counterstained with DAPI to demonstrate the overall cellular anatomy of the region (**F**). *CD11b: green; DAPI: blue. Scale bars: 200 μm (***A**,**B**), 100 μm (**C**-**F**).

In contrast to ED-1, CD11b stains all microglia regardless of activation state. When the microglia transform into activated macrophages they also undergo a morphological transformation from a resting ramified state to an amoeboid state. Figure 4 demonstrates that this morphological shift only occurred in areas of cellular damage. There were no amoeboid shaped microglia detected on the contralateral side (Figure 4A,B). Ipsilaterally, both microglial morphologies were observed. Ramified microglia were seen in subcortical regions that did not exhibit cellular damage (Figure 4C) and amoeboid microglia were present at the site of impact (Figure 4D) and in subcortical regions where cellular damage had occurred. This duality can be seen definitively in the ipsilateral hippocampal region where there is cellular damage interspersed between structurally intact tissue (Figure 4E,F). No immunostaining was seen in the cortex or hippocampus in negative control sections (data not shown).

### Inflammatory gene expression patterns

**Figure 5:**
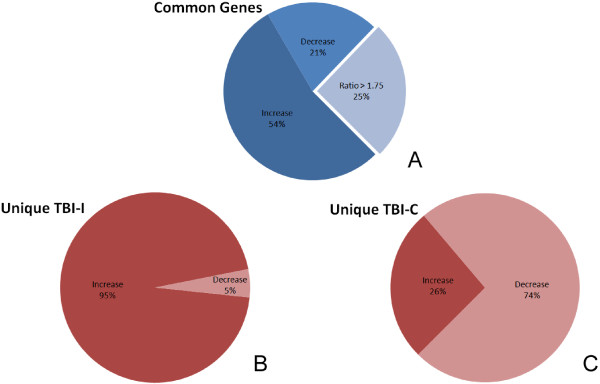
**Breakdown of IR genes based on up- and downregulation in expression. **(**A**) There were 146 genes that change more than 2-fold on both sides of the brain. Seventy-five percent of them (109 genes) changed similarly while the remaining 25% (37 genes) changed differently (ratio >1.75; see text). (**B**) 188 IR genes changed uniquely on the ipsilateral side of the brain and 95% (179 genes) of those increased in expression. (**C**) 38 IR genes changed uniquely on the contralateral side of the brain and 74% (28 genes) of those decreased in expression.

Focusing on the inflammatory response genes in our datasets, we determined that 372 IR genes had a greater than 2-fold change in expression. Of these genes, 146 genes changed on both the ipsilateral and contralateral sides of the brain. In order to determine whether these common genes changed differently on one side of the brain compared to the other, we calculated the ratio of the TBI-I fold change to the TBI-C (contralateral vs naïve) fold change. Those genes that had a TBI-I/TBI-C ratio greater than 1.75 were determined to have change differently. We observed that 109 of the common IR genes (75%) changed similarly (TBI-I/TBI-C ratio < 1.75; Figure 5A). Of the genes that changed similarly, 79 genes (54%) increased in expression and 30 genes (21%) decreased in expression. The remaining 37 common IR genes (25%) were changed differently (TBI-I/TBI-C ratio > 1.75) (Figure 5A). Table 2 shows the 37 common IR genes that change differently. These genes span all cellular compartments (extracellular space, plasma membrane, cytoplasm, and nucleus) with diverse molecule types. The expression of all these genes was lower on the contralateral side of the brain. Because of their different expression patterns, these genes became our first group of genes of interest (GOI). Notable genes identified included CCND1, SPP1, ERAP1, LYN, THRA, TIMP1, the transcription regulators STAT3, CEBPD, and CBL, and the plasma membrane receptors and signaling molecules IL6ST, CD44, EGFR, ITGA5, and SDC1.

**Table 2:** Genes that change differently on each side of the brain

**Gene symbol**	**Entrez gene name**	**TBI-I fold change**	**TBI-C fold change**	**TBI-I/TBI-C ratio**	**Molecule type**
***Extracellular space***					
CSF1	colony stimulating factor 1 (macrophage)	3.70	2.09	1.77	cytokine
SPP1	secreted phosphoprotein 1	37.91	2.37	15.99	cytokine
ERAP1	endoplasmic reticulum aminopeptidase 1	5.84	3.05	1.92	peptidase
LCN2	lipocalin 2	71.82	3.90	18.44	transporter
TGFB2	transforming growth factor, beta 2	-4.00	-7.97	1.99	growth factor
SERPINA3	serpin peptidase inhibitor, clade A (alpha-1 antiproteinase, antitrypsin), member 3	58.49	2.51	23.31	other
SERPING1	serpin peptidase inhibitor, clade G (C1 inhibitor), member 1	5.81	2.03	2.86	other
TIMP1	TIMP metallopeptidase inhibitor 1	38.49	2.10	18.32	other
***Plasma membrane***					
HLA-C	major histocompatibility complex, class I, C	9.30	3.66	2.54	transmembrane receptor
IGSF6	immunoglobulin superfamily, member 6	22.46	3.27	6.87	transmembrane receptor
IL13RA1	interleukin 13 receptor, alpha 1	4.53	2.27	1.99	transmembrane receptor
IL6ST	interleukin 6 signal transducer (gp130, oncostatin M receptor)	2.31	-3.28	7.57	transmembrane receptor
THBD	thrombomodulin	3.85	2.09	1.84	transmembrane receptor
KCNN4	potassium intermediate/small conductance calcium-activated channel, subfamily N, member 4	3.09	-9.43	29.12	ion channel
EGFR	epidermal growth factor receptor	6.77	2.37	2.85	kinase
MGLL	monoglyceride lipase	-7.85	-18.15	2.31	enzyme
CD44	CD44 molecule (Indian blood group)	15.56	2.40	6.49	other
CLEC12A	C-type lectin domain family 12, member A	10.92	2.15	5.08	other
ITGA5	integrin, alpha 5 (fibronectin receptor, alpha polypeptide)	4.83	2.68	1.80	other
SDC1	syndecan 1	13.68	2.57	5.33	other
***Cytoplasm***					
LYN	v-yes-1 Yamaguchi sarcoma viral related oncogene homolog	6.94	3.78	1.84	kinase
PDE4B	phosphodiesterase 4B, cAMP-specific	5.60	2.36	2.37	enzyme
PTPN4	protein tyrosine phosphatase, non-receptor type 4 (megakaryocyte)	4.49	2.21	2.03	phosphatase
RASA1	RAS p21 protein activator (GTPase activating protein) 1	2.39	-2.11	5.04	transporter
HSPB1	heat shock 27 kDa protein 1	46.92	2.64	17.78	other
LCP1	lymphocyte cytosolic protein 1 (L-plastin)	6.08	2.80	2.17	other
LSP1	lymphocyte-specific protein 1	11.72	2.14	5.47	other
MYO1F	myosin IF	4.27	2.26	1.89	other
***Nucleus***					
CBL	Cas-Br-M (murine) ecotropic retroviral transforming sequence	-3.40	-6.13	1.80	transcription regulator
CEBPD	CCAAT/enhancer binding protein (C/EBP), delta	11.27	2.04	5.53	transcription regulator
DEK	DEK oncogene	-3.01	-7.35	2.45	transcription regulator
STAT3	signal transducer and activator of transcription 3 (acute-phase response factor)	4.22	-3.77	15.91	transcription regulator
MX1	myxovirus (influenza virus) resistance 1, interferon-inducible protein p78 (mouse)	28.18	7.33	3.85	enzyme
TOP2A	topoisomerase (DNA) II alpha 170 kDa	2.26	-2.41	5.44	enzyme
THRA	thyroid hormone receptor, alpha	-2.80	-11.52	4.12	ligand-dependent nuclear receptor
CCND1	cyclin D1	2.15	-2.03	4.36	other
***Unknown***					
Slpi (includes others)	secretory leukocyte peptidase inhibitor	82.91	3.12	26.58	other

TBI-I/TBI-C Ratio: Gene increased on both sides: ratio = (TBI-I)/(TBI-C); Gene decreased on both sides: ratio = 1/[(TBI-I)/(TBI-C)];

Gene increased ipsilaterally and decreased contralaterally: ratio = (TBI-I)/-[1/(TBI-C)].

There were 188 IR genes that changed uniquely on the ipsilateral side of the brain. 179 of those genes (95%) increased while 9 genes (5%) decreased in expression (Figure 5B). Only 38 IR genes change uniquely on the contralateral side of the brain and, in contrast to what we observed on the ipsilateral side, only 10 genes (26%) increased while 28 genes (74%) decreased in expression (Figure 5C).

### Canonical pathway analysis

**Figure 6:**
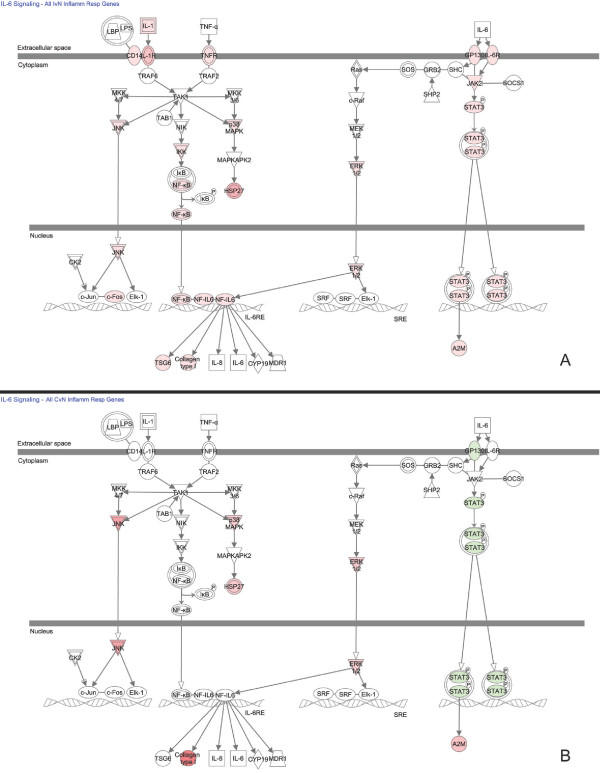
**Canonical pathway analysis. **The IL-6 signaling pathway showing the relative expression values for all TBI-I IR genes (**A**) and all TBI-C IR genes (**B**) involved in this pathway. *red: relative increase in expression; green: relative decrease in expression; white: no change in expression*.

We used canonical pathway and network analysis in IPA to identify genes in our datasets that were most relevant to the observed IR. Because IPA displays only the relative expression values, we defined GOI, in this context, as those genes that either increased or decreased on one side of the brain and showed no change in expression on the other side or genes that had opposite changes in expression. These genes were easily identified by side-by-side comparison of the canonical pathway and gene networks overlaid with the expression values of the TBI-I and TBI-C IR datasets. Canonical pathways in IPA are well-characterized metabolic and cell signaling pathways derived from information found in specific journal articles, review articles, text books, and KEGG Ligand [11]. Figure 6 shows the IL-6 signaling canonical pathway. IPA determined that this pathway was highly associated with the TBI-I dataset. Additionally, this pathway also includes elements of IL-1, TNF-α, and lipopolysaccharide (LPS) signaling. IL-1 and TNF-α were previously associated with TBI and inflammation [9,12-14] and TBI induced inflammation has been shown to be exacerbated by LPS challenge [15]. By overlaying the relative expression values for all TBI-I IR genes (Figure 6A) and all TBI-C IR genes (Figure 6B), we were able to identify a number of GOI that were increased in TBI-I and were either unchanged or decreased in TBI-C, including IL1B, several transmembrane cytokine receptors and the transcriptional regulators NFkB, STAT3, CEBPB (NF-IL6), and FOS.

### Gene network analysis

**Figure 7:**
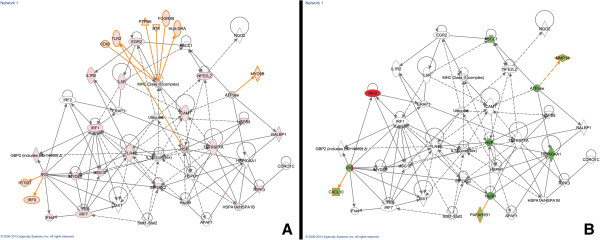
**Analysis of the highest scored IPA generated gene network. **This was the highest scored gene network associated with the TBI-I/TBI-C union dataset. The relative expression values of the unique TBI-I IR genes (**A**) and unique TBI-C IR genes (**B**) included in this network are shown. *red: relative increase in expression; green: relative decrease in expression; white: no change in expression; orange connections and outlines: direct connections with gene groups and complexes in the original network.*

In contrast to canonical pathways, which are relatively immutable in IPA, gene networks are generated *de novo* in IPA based on the list of genes that are imported. IPA takes “seed” molecules from the gene list, searches the Ingenuity Knowledge Base, and uses a network algorithm to draw connections between molecules based on biological function [16]. In order to generate networks that included IR genes that changed on both sides of the brain, we combined the TBI-I and TBI-C datasets and performed an IPA core analysis on that union dataset. IPA scores the networks in order to rank them according to their degree of relevance to the network eligible molecules in your dataset [16]. Figure 7 shows the highest scored network associated with our union dataset. For this network, we overlaid the relative expression values for the unique TBI-I IR genes (Figure 7A) and unique TBI-C IR genes (Figure 7B) and were able to identify 29 GOI, such as the chemokine CXCL10, a number of cytokine and toll-like receptors, heat shock proteins, and transcriptional regulators that were increased in TBI-I and were either unchanged or decreased in TBI-C. Interestingly, IRF2 was upregulated on the contralateral side of the brain.

**Figure 8:**
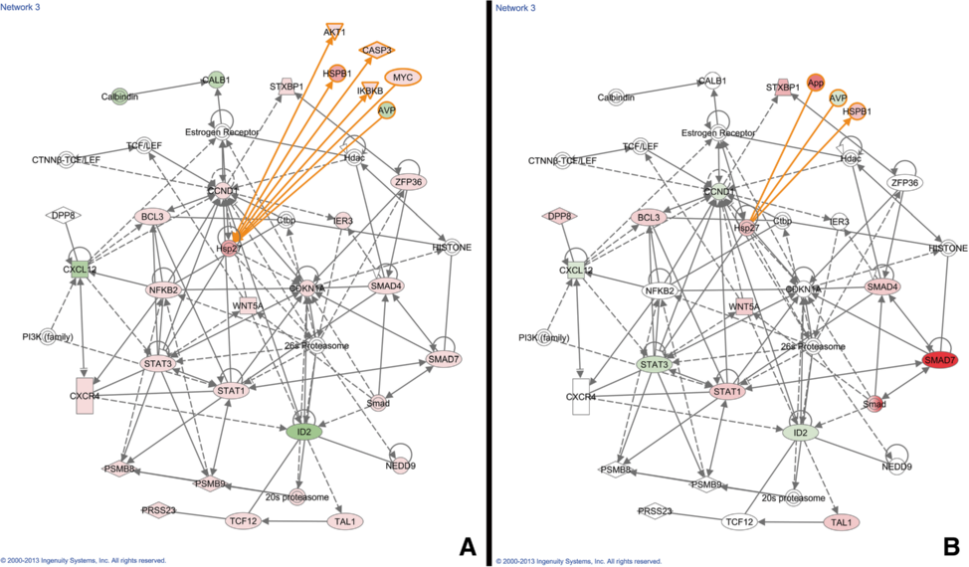
**Analysis of a highly scored IPA generated gene network. **This gene network was scored in the top three networks associated with the TBI-I/TBI-C union dataset. The relative expression values of all TBI-I IR genes (**A**) and all TBI-C IR genes (**B**) included in this network are shown. *red: relative increase in expression; green: relative decrease in expression; white: no change in expression; orange connections and outlines: direct connections with gene groups and complexes in the original network.*

Figure 8 shows another network that was scored in the top three networks associated with our union dataset. For this network, we overlaid the relative expression values for all TBI-I IR genes (Figure 8A) and all TBI-C IR genes (Figure 8B) and were able to identify 15 GOI including the cytokine CXCL1, a number of genes in the JAK/STAT pathway, NFkB and several genes associated with apoptosis.

**Figure 9:**
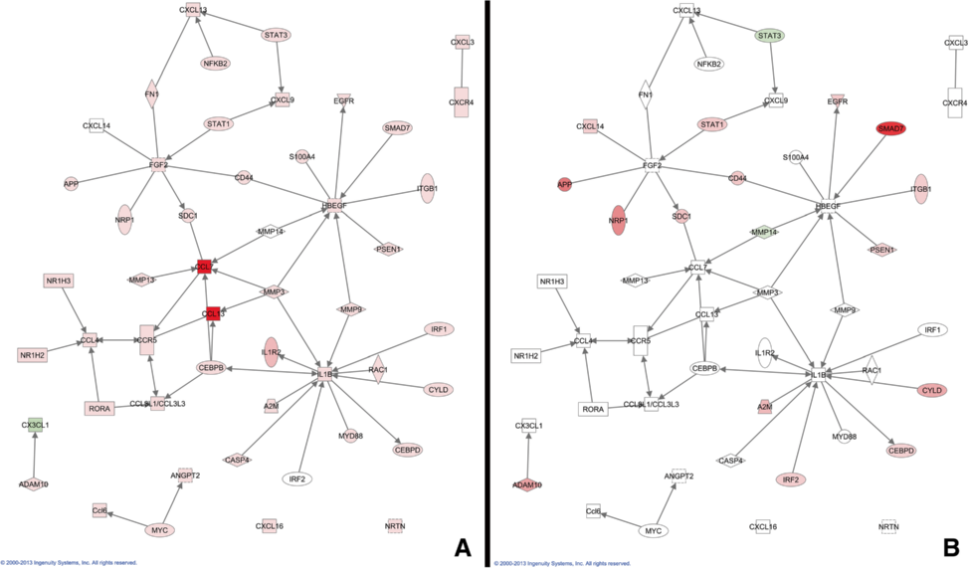
**Cytokine and growth factor network analysis. **The gene network was created in IPA by seeding with the IR cytokines and growth factors expressed uniquely in the TBI-I analysis and “growing” those genes into a network by showing their direct connections with genes in the union of all TBI-I and TBI-C IR genes dataset. The relative expression values of all TBI-I IR genes (**A**) and all TBI-C IR genes (**B**) included in this network are shown. *red: relative increase in expression; green: relative decrease in expression; white: no change in expression.*

Because the IR is, in part, humoral in nature, we created a network within IPA by seeding it with IR cytokines and growth factors expressed uniquely in the TBI-I dataset and “growing them” (making direct functional connections) to the TBI-I/TBI-C union dataset. Figure 9 shows the resultant network. By overlaying the relative expression values for all TBI-I IR genes (Figure 9A) and all TBI-C IR genes (Figure 9B), we were able to identify 23 more GOI. Notable genes were IL1B, chemokines CCL7, CCL13, CXCL13 and CCL4, peptidases MMP13, MMP3, and MMP9 and many inflammation associated transcription factors.

### Compiling the gene interaction hierarchy (GIH)

By combining the GOI identified through canonical pathway and network analysis with those in Table 2, we identified a total of 114 GOI. In order to determine which genes might be most relevant in the IR, we ranked these genes relative to each other by the number of direct interactions each had with the other GOI. Our analysis showed that 95 of the GOI formed an interconnected network, leaving 19 “orphan” genes (see Additional file 1). Genes having more than 10 connections (1^st^ order) within the main GOI network were considered “primary” in this analysis (see Additional file 2 for an example). Genes having 5–10 connections were considered “secondary” (see Additional file 3 for an example) and those with less than 5 connections were considered “peripheral”. The resultant GIH is displayed in Table 3.

**Table 3:** Gene interaction hierarchy (GIH)

**Gene symbol**	**Entrez gene name**	**Fold change (TBI-I | TBI-C)**	**Cellular compartment**	**Molecular type**
**Primary**				
CEBPB	CCAAT/enhancer binding protein (C/EBP), beta	3.37 | ~	Nucleus	transcription regulator
FOS	FBJ murine osteosarcoma viral oncogene homolog	2.83 | ~	Nucleus	transcription regulator
IRF1	interferon regulatory factor 1	2.22 | ~	Nucleus	transcription regulator
IRF7	interferon regulatory factor 7	2.78 | ~	Nucleus	transcription regulator
MYC	v-myc myelocytomatosis viral oncogene homolog (avian)	3.96 | ~	Nucleus	transcription regulator
NFKB2	nuclear factor of kappa light polypeptide gene enhancer in B-cells 2 (p49/p100)	2.77 | ~	Nucleus	transcription regulator
*STAT3*	signal transducer and activator of transcription 3 (acute-phase response factor)	4.22 | -3.77	Nucleus	transcription regulator
*EGFR*	epidermal growth factor receptor	6.77 | 2.37	Plasma membrane	kinase
IKBKB	inhibitor of kappa light polypeptide gene enhancer in B-cells, kinase beta	2.13 | ~	Cytoplasm	kinase
JAK2	Janus kinase 2	2.53 | ~	Cytoplasm	kinase
*LYN*	v-yes-1 Yamaguchi sarcoma viral related oncogene homolog	6.94 | 3.78	Cytoplasm	kinase
FN1	fibronectin 1	3.97 | ~	Extracellular space	enzyme
**HSP90AA1**	heat shock protein 90 kDa alpha (cytosolic), class A member 1	~ | -4.84	Cytoplasm	enzyme
IL1B	interleukin 1, beta	5.17 | ~	Extracellular space	cytokine
CASP3	caspase 3, apoptosis-related cysteine peptidase	2.54 | ~	Cytoplasm	peptidase
TLR4	toll-like receptor 4	2.70 | ~	Plasma membrane	transmembrane receptor
*CCND1*	cyclin D1	2.15 | -2.03	Nucleus	other
*CD44*	CD44 molecule (Indian blood group)	15.56 | 2.40	Plasma membrane	other
CDKN1A	cyclin-dependent kinase inhibitor 1A (p21, Cip1)	2.68 | ~	Nucleus	other
MYD88	myeloid differentiation primary response gene (88)	2.70 | ~	Plasma membrane	other
**Secondary**				
*ERAP1*	endoplasmic reticulum aminopeptidase 1	5.84 | 3.05	Extracellular space	peptidase
MMP13	matrix metallopeptidase 13 (collagenase 3)	2.26 | ~	Extracellular space	peptidase
**MMP14**	matrix metallopeptidase 14 (membrane-inserted)	~ | -3.03	Extracellular space	peptidase
MMP3	matrix metallopeptidase 3 (stromelysin 1, progelatinase)	4.05 | ~	Extracellular space	peptidase
MMP9	matrix metallopeptidase 9 (gelatinase B, 92 kDa gelatinase, 92 kDa type IV collagenase)	7.18 | ~	Extracellular space	peptidase
PSMB8	proteasome (prosome, macropain) subunit, beta type, 8 (large multifunctional peptidase 7)	3.30 | ~	Cytoplasm	peptidase
PSMB9	proteasome (prosome, macropain) subunit, beta type, 9 (large multifunctional peptidase 2)	2.32 | ~	Cytoplasm	peptidase
ICAM1	intercellular adhesion molecule 1	2.60 | ~	Plasma membrane	transmembrane receptor
IL6R	interleukin 6 receptor	2.32 | ~	Plasma membrane	transmembrane receptor
*IL6ST*	interleukin 6 signal transducer (gp130, oncostatin M receptor)	2.31 | -3.28	Plasma membrane	transmembrane receptor
TLR2	toll-like receptor 2	2.33 | ~	Plasma membrane	transmembrane receptor
TNFRSF1A	tumor necrosis factor receptor superfamily, member 1A	3.56 | ~	Plasma membrane	transmembrane receptor
*CBL*	Cas-Br-M (murine) ecotropic retroviral transforming sequence	-3.40 | -6.13	Nucleus	transcription regulator
*CEBPD*	CCAAT/enhancer binding protein (C/EBP), delta	11.27 | 2.04	Nucleus	transcription regulator
**IRF2**	interferon regulatory factor 2	~ | 2.17	Nucleus	transcription regulator
IRF9	interferon regulatory factor 9	3.26 | ~	Nucleus	transcription regulator
CCL7	chemokine (C-C motif) ligand 7	124.78 | ~	Extracellular space	cytokine
**CXCL10**	chemokine (C-X-C motif) ligand 10	~ | -3.29	Extracellular space	cytokine
*SPP1*	secreted phosphoprotein 1	37.91 | 2.37	Extracellular space	cytokine
AKT1	v-akt murine thymoma viral oncogene homolog 1	2.04 | ~	Cytoplasm	kinase
BTK	Bruton agammaglobulinemia tyrosine kinase	2.29 | ~	Cytoplasm	kinase
EIF2AK2	eukaryotic translation initiation factor 2-alpha kinase 2	2.18 | ~	Cytoplasm	kinase
CCR5	chemokine (C-C motif) receptor 5	2.09 | ~	Plasma membrane	G-protein coupled receptor
CXCR4	chemokine (C-X-C motif) receptor 4	2.31 | ~	Plasma membrane	G-protein coupled receptor
HBEGF	heparin-binding EGF-like growth factor	7.07 | ~	Extracellular space	growth factor
FGF2	fibroblast growth factor 2 (basic)	2.39 | ~	Extracellular space	growth factor
RORA	RAR-related orphan receptor A	2.50 | ~	Nucleus	ligand-dependent nuclear receptor
*THRA*	thyroid hormone receptor, alpha	-2.80 | -11.52	Nucleus	ligand-dependent nuclear receptor
RAC1	ras-related C3 botulinum toxin substrate 1 (rho family, small GTP binding protein Rac1)	2.32 | ~	Plasma membrane	enzyme
PTPN6	protein tyrosine phosphatase, non-receptor type 6	3.64 | ~	Cytoplasm	phosphatase
HSPA1A/HSPA1B	heat shock 70 kDa protein 1A	3.14 | ~	Cytoplasm	other
*ITGA5*	integrin, alpha 5 (fibronectin receptor, alpha polypeptide)	4.83 | 2.68	Plasma membrane	other
*SDC1*	syndecan 1	13.68 | 2.57	Plasma membrane	other
*TIMP1*	TIMP metallopeptidase inhibitor 1	38.49 | 2.10	Extracellular space	other
**Peripheral**				
CCL13/CCL2	chemokine (C-C motif) ligand 13/2	195.46 | ~	Extracellular space	cytokine
CCL3L1/CCL3L3	chemokine (C-C motif) ligand 3-like 1	5.27 | ~	Extracellular space	cytokine
CCL4	chemokine (C-C motif) ligand 4	2.16 | ~	Extracellular space	cytokine
Ccl6	chemokine (C-C motif) ligand 6	10.29 | ~	Extracellular space	cytokine
*CSF1*	colony stimulating factor 1 (macrophage)	3.70 | 2.09	Extracellular space	cytokine
CXCL13	chemokine (C-X-C motif) ligand 13	3.78 | ~	Extracellular space	cytokine
**CXCL14**	chemokine (C-X-C motif) ligand 14	~ | 2.13	Extracellular space	cytokine
CXCL9	chemokine (C-X-C motif) ligand 9	2.85 | ~	Extracellular space	cytokine
CD14	CD14 molecule	10.66 | ~	Plasma membrane	transmembrane receptor
FCGR2B	Fc fragment of IgG, low affinity IIb, receptor (CD32)	5.12 | ~	Plasma membrane	transmembrane receptor
*HLA-C*	major histocompatibility complex, class I, C	9.30 | 3.66	Plasma membrane	transmembrane receptor
HLA-DRA	major histocompatibility complex, class II, DR alpha	2.70 | ~	Plasma membrane	transmembrane receptor
*IL13RA1*	interleukin 13 receptor, alpha 1	4.53 | 2.27	Plasma membrane	transmembrane receptor
IL1R2	interleukin 1 receptor, type II	37.03 | ~	Plasma membrane	transmembrane receptor
GBP2 (includes EG:14469)	guanylate binding protein 2, interferon-inducible	9.13 | ~	Cytoplasm	enzyme
*MX1*	myxovirus (influenza virus) resistance 1, interferon-inducible protein p78 (mouse)	28.18 | 7.33	Nucleus	enzyme
RALBP1	ralA binding protein 1	2.23 | ~	Cytoplasm	enzyme
*TOP2A*	topoisomerase (DNA) II alpha 170 kDa	2.26 | -2.41	Nucleus	enzyme
EGR2	early growth response 2	2.27 | ~	Nucleus	transcription regulator
NFE2L2	nuclear factor (erythroid-derived 2)-like 2	2.45 | ~	Nucleus	transcription regulator
ZFP36	zinc finger protein 36, C3H type, homolog (mouse)	7.28 | ~	Nucleus	transcription regulator
**ABCC1**	ATP-binding cassette, sub-family C (CFTR/MRP), member 1	~ | -3.11	Plasma membrane	transporter
*LCN2*	lipocalin 2	71.82 | 3.90	Extracellular space	transporter
*RASA1*	RAS p21 protein activator (GTPase activating protein) 1	2.39 | -2.11	Cytoplasm	transporter
NR1H2	nuclear receptor subfamily 1, group H, member 2	2.15 | ~	Nucleus	ligand-dependent nuclear receptor
NR1H3	nuclear receptor subfamily 1, group H, member 3	2.16 | ~	Nucleus	ligand-dependent nuclear receptor
HSPB8	heat shock 22 kDa protein 8	4.11 | ~	Cytoplasm	kinase
RIPK3	receptor-interacting serine-threonine kinase 3	7.55 | ~	Plasma membrane	kinase
CASP4	caspase 4, apoptosis-related cysteine peptidase	3.05 | ~	Cytoplasm	peptidase
**DPP8**	dipeptidyl-peptidase 8	~ | 2.06	Cytoplasm	peptidase
ANGPT2	angiopoietin 2	2.98 | ~	Extracellular space	growth factor
CD63	CD63 molecule	2.07 | ~	Plasma membrane	other
*HSPB1*	heat shock 27 kDa protein 1	46.92 | 2.64	Cytoplasm	other
IER3	immediate early response 3	2.35 | ~	Cytoplasm	other
IFI44	interferon-induced protein 44	3.12 | ~	Cytoplasm	other
IFITM3	interferon induced transmembrane protein 3	3.48 | ~	Plasma membrane	other
ISG15	ISG15 ubiquitin-like modifier	3.53 | ~	Extracellular space	other
NEDD9	neural precursor cell expressed, developmentally down-regulated 9	2.59 | ~	Nucleus	other
S100A4	S100 calcium binding protein A4	4.73 | ~	Cytoplasm	other
*SERPINA3*	serpin peptidase inhibitor, clade A (alpha-1 antiproteinase, antitrypsin), member 3	58.49 | 2.51	Extracellular space	other
TNFAIP6	tumor necrosis factor, alpha-induced protein 6	3.02 | ~	Extracellular space	other
**Orphan**				
*MGLL*	monoglyceride lipase	-7.85 | -18.15	Plasma membrane	enzyme
MYO9B	myosin IXB	2.18 | ~	Cytoplasm	enzyme
**PAFAH1B1**	platelet-activating factor acetylhydrolase 1b, regulatory subunit 1 (45 kDa)	~ | -2.86	Cytoplasm	enzyme
*PDE4B*	phosphodiesterase 4B, cAMP-specific	5.60 | 2.36	Cytoplasm	enzyme
*DEK*	DEK oncogene	-3.01 | -7.35	Nucleus	transcription regulator
TCF12	transcription factor 12	2.16 | ~	Nucleus	transcription regulator
*IGSF6*	immunoglobulin superfamily, member 6	22.46 | 3.27	Plasma membrane	transmembrane receptor
*THBD*	thrombomodulin	3.85 | 2.09	Plasma membrane	transmembrane receptor
*TGFB2*	transforming growth factor, beta 2	-4.00 | -7.97	Extracellular space	growth factor
*KCNN4*	potassium intermediate/small conductance calcium-activated channel, subfamily N, member 4	3.09 | -9.43	Plasma membrane	ion channel
PRSS23	protease, serine, 23	4.05 | ~	Extracellular space	peptidase
*PTPN4*	protein tyrosine phosphatase, non-receptor type 4 (megakaryocyte)	4.49 | 2.21	Cytoplasm	phosphatase
CALB1	calbindin 1, 28 kDa	-2.09 | ~	Cytoplasm	other
*CLEC12A*	C-type lectin domain family 12, member A	10.29 | 2.15	Plasma membrane	other
*LCP1*	lymphocyte cytosolic protein 1 (L-plastin)	6.08 | 2.80	Cytoplasm	other
*LSP1*	lymphocyte-specific protein 1	11.72 | 2.14	Cytoplasm	other
*MYO1F*	myosin IF	4.27 | 2.26	Cytoplasm	other
*SERPING1*	serpin peptidase inhibitor, clade G (C1 inhibitor), member 1	5.81 | 2.03	Extracellular space	other
*Slpi (includes others)*	secretory leukocyte peptidase inhibitor	82.91 | 3.12	unknown	other

Primary: >10 connections in GOI network (see text); Secondary: 5–10 connections in GOI network;

Peripheral: <5 connections in GOI network; Orphan: No connections in GOI network;

Italics= > Gene changes on both sides of the brain; Bold= > Gene changes only contralaterally; All other genes change only ipsilaterally;

## Discussion

In this study, we used microarray technology to examine cellular and molecular mechanisms associated with secondary brain injury following TBI. Our findings indicated that the inflammatory response and its associated genes and pathways are significant in the post-TBI molecular response. This is consistent with our published studies indicating that inflammation is involved with delayed, secondary neuronal injury following other acute brain injuries (ABI’s) including stroke and neurotoxin exposure [17-20]. Activated microglia, astrocytes and macrophages have been shown to be the source of several of the inflammatory molecules identified in this study [10,21-23]. The presence of activated inflammatory cells ipsilaterally and their absence contralaterally was confirmed by immunohistochemistry. This cellular inflammation offers further evidence for pro-inflammatory molecules being produced in the region of tissue damage following TBI.

Despite the absence of activated inflammatory cells on the contralateral side of the brain, our results also showed that TBI resulted in a significant alteration of the inflammatory gene response on the both sides of the brain. The observed IR gene expression pattern suggests that there is a baseline IR throughout the whole brain due to unilateral CCI. This is demonstrated by the nearly 30% of all IR genes that change similarly on both sides of the brain (109 of 372 IR genes). Above this baseline of inflammation, a distinct IR gene expression pattern emerges. The expression level is higher on the ipsilateral side for all the remaining genes expressed on both sides of the brain. 95% of the IR genes expressed only in ipsilateral tissues increase in expression while 74% of the IR genes expressed only in contralateral tissues decrease in expression. Examination of the contralateral gene expression in our GIH showed that 11 genes (3 primary tier, 3 secondary tier, 3 peripheral tier and 2 orphan) decreased in expression contralaterally while either remaining unchanged or increasing in expression ipsilaterally. Only 3 genes (1 secondary and 2 peripheral tier) increased contralaterally and remained unchanged ipsilaterally. This demonstrated that the most significant contralateral IR genes in this analysis show decreased expression.

Taken together with the cellular inflammation data, we can surmise that increased expression of the majority of IR genes ipsilaterally results in the development of functional inflammation that can contribute to secondary neural injury. Similarly, it is likely that the suppression of the majority of IR genes contralaterally prevents this development in brain regions remote to the injury. This summary of the overall IR gene expression does not take into account that some IR genes may be classified as anti-inflammatory in certain contexts. However, any counter effects of anti-inflammatory molecules seems to be negligible as the cellular inflammation pattern follows the IR gene expression pattern assuming the majority of IR genes are pro-inflammatory. The contralateral inflammatory response without detectable cellular inflammation also fits well with the idea that some inflammation may actually be beneficial following TBI [9].

Through canonical pathway and network analysis combined with identification of the common genes that change differently on each side of the brain, we identified 114 GOI. Many of these genes have been previously associated with the IR following ABI’s (i.e., TBI, stroke, nerve agent exposure) including CCL13/CCL2, CCL4, CCL6, CCL7, CCR5, CD14, CD44, CDKN1A, CEBPB, CEBPD, CXCL13, CXCL9, CSF1, FOS, HBEGF, HSPA1A/HSPA1B, ICAM1, IER3, IL-1β, IRF1, IRF2, JAK2, LCN2, MMP3, MMP9, MYD88, NF-κB, PSMB8, S100A4, SPP1, STAT3, TLR2, TLR4, and TNFRSF1A [10,13,21,24-34]. These results support the utility of our methods for identifying the significant genes related to this biological function. Additionally, several IR genes that appear to be novel in the context of TBI were also identified, providing new targets for future study. These genes included HSP90AA1, ERAP1, PSMB9, CBL, BTK, RORA, THRA, and ITGA5. We wanted to take our analysis one step further and determine which genes were likely the most critical in the observed IR. We accomplished this by creating a network of these genes and determining how many 1^st^ order connections each gene had with the other genes in the network. A GIH was created based on these numbers and there were some intriguing findings in terms of which tier certain molecule types predominated.

Not surprisingly, a large number of transcription regulators were included in the primary and secondary tiers as these molecules are the point of convergence for many of the inflammatory pathways and the regulatory step in the production of new proteins. Somewhat surprising was the large number of cytokines that fell into the peripheral tier since considerable focus has been placed on cytokines as mediators of inflammation and targets for therapeutic intervention [10,21,24]. This result may be due to a near 1-to-1 relationship that these cytokines have with their receptors, limiting the 1^st^ order connections in the GOI network. While cytokines clearly play an important role in initiating the IR, they may not be the most critical molecules in modulating the IR. Only one cytokine, IL1B, is in the primary tier of our hierarchy while CCL7, CXCL10, and SPP1 are in the secondary tier. This suggests that neuroprotective strategies directed at one of the lower tier cytokines may not be sufficient for limiting the IR [35]. Further, while therapeutic hypothermia after TBI has been shown to improve outcome, inflammatory cytokine levels were unaffected, implying minimal cytokine involvement in the observed neuroprotection [26]. However, targeting the cytokines in the higher tiers may produce effective modification of the IR [36]. Our GIH shows that regulation of transcription, phosphorylation (kinases), extracellular matrix/cell adhesion (FN1, MMPs, ICAM1), and receptors (transmembrane and G-coupled) figure prominently (primary and secondary tiers) in the post-TBI IR. These molecules may, therefore, be more efficient targets for therapeutic strategies to combat post-TBI inflammation because they are activated regardless of the initiating factor.

Two well characterized signaling pathways stood out in our GIH: toll-like receptor/NF-κB signaling and JAK/STAT signaling. There was some concern that our choice of canonical pathway may have skewed our analysis towards these signaling pathways (toll-like receptor/NF-κB and JAK/STAT) because large portions of both of these pathways are included in IL-6 signaling (Figure 4). While 12 GOI are included in that pathway, 8 of these genes were also identified by other analyses used to identify GOI, further supporting their importance to the post-TBI IR. Additionally, interconnection of the genes in IL-6 signaling could not account for the total number of 1^st^ order connections for these genes in the GOI network used to build the GIH. This was especially true for genes in the primary tier. 19 GOI were either a part of (CEBPB, FOS, NFKB2, EGFR, IKBKB, IL1B, TLR4, MYD88, TLR2, TNFRSF1A, CD14, IL1R2), a product of (IRF1, FN1, CCND1, CASP3), or associated with (IRF7, LYN, HSP90AA1) toll-like receptor/NF-κB signaling [8]. 15 of these genes were in the primary tier of the GIH. Secondarily, 5 GOI (JAK2, STAT3, CDKN1A, IL6R, IL6ST) were a part of JAK/STAT signaling [8], and three of these genes were in the primary tier. Based on our analysis, these signaling pathways are likely candidates for the induction of post-TBI IR. Therefore, therapeutic strategies aimed at the molecules in these pathways may reduce TBI-induced inflammation and, by extension, post-TBI neuronal death.

It should be noted that this study only takes a snapshot at 24 hours post-injury of a dynamic and evolving molecular process initiated by TBI [37]. Therefore, significant molecular events that precede and follow this time point are not reflected in these data. For a more complete examination of the molecular response to TBI, a study including multiple time points before and after 24 hours will be necessary and are underway in our laboratory. Further, confirmation of the biological relevance of any observed gene expression profile following TBI is a critical next step to exploring potential therapies for brain trauma [26,38].

## Conclusions

Microarray analysis is a powerful tool that allows for the analysis of thousands of genes simultaneously. We demonstrated that TBI was associated with a powerful pro-inflammatory response in ipsilateral brain tissues. We also noted the distinct IR gene expression pattern that suggests a remote anti-inflammatory response. The use of multiple network and pathway analyses to identify GOI aided in making our datasets manageable and revealed 2 distinct pathways, toll-like receptor/NF-κB signaling and JAK/STAT signaling, associated with post-TBI secondary neural injury. Our GIH provides a starting point for investigating therapeutic targets in a ranked order that is somewhat different than what has been presented previously in microarray studies. In addition to being a vehicle for identifying potential targets for post-TBI therapeutic strategies, our GIH can also provide a context for evaluating the potential of therapeutic agents currently in development.

## Methods

### Animals

All animals used in these studies were treated humanely and with regard for alleviation of suffering and pain and all protocols involving animals were approved by the IACUCs of Morehouse School of Medicine and/or The Georgia Institute of Technology prior to the initiation of experimentation. Adult male Sprague–Dawley rats (290-300 g; Charles River Laboratories International, Inc., USA) were housed individually in standard plastic cages in a temperature-controlled room (22 ± 2°C) on a 12 h reverse light–dark cycle. Food and water were provided ad libitum.

### Controlled cortical impact

Under isoflurane anesthesia, rats received a unilateral controlled cortical impact (CCI/TBI) using the Pittsburgh Precision Instruments, Inc. device. A craniotomy was made with the center 4 mm posterior and 3–4 mm lateral to bregma using a 6 mm diameter trephan drill bit. The impact was done at an angle of 15° from vertical with a velocity of 3 m/s to a depth of 2 mm using a 5 mm diameter impact tip. The rats were sacrificed 24 h post-injury and the brains were removed for RNA isolation or histology.

### RNA preparation and GeneChip analysis

The ipsilateral hemi-brain tissue at the site of the injury, the corresponding contralateral hemi-brain tissue, and naïve (control) brain tissue (n = 3 for each) were used for RNA isolation. Total RNA was extracted with TRIzol Reagent (Life Technologies, Rockville, MD, USA) and cleaned (RNAqueous Kit, Ambion, Austin, TX, USA). The RNA was prepared for microarray hybridization with the GeneChip^®^ 3^′^ IVT Express Kit (Affymetrix Inc., Santa Clara, CA, USA) aRNA amplification procedure. Briefly, total RNA was reverse transcribed to synthesize first-strand cDNA containing a T7 promoter sequence. The single-stranded cDNA was converted into a double-stranded DNA template for transcription. The reaction employed DNA polymerase and RNase H to simultaneously degrade the RNA and synthesize second-strand cDNA. *In vitro* transcription generated multiple copies of biotin-modified aRNA from the double-stranded cDNA templates (this was the amplification step). aRNA Purification removed unincorporated NTPs, salts, enzymes, and inorganic phosphate to improve the stability of the biotin-modified aRNA. Finally, the labeled aRNA was fragmented to prepare the target for hybridization to GeneChip^®^ 3^′^ expression arrays [39]. Following fragmentation, 15 μg of the biotinylated cRNA was hybridized to an Affymetrix Rat Genome 230 2.0 GeneChip. The chips were hybridized at 45°C for 16 h, and then washed, stained with streptavidin–phycoerythrin and scanned according to manufacturing guidelines.

### Microarray data analysis

Data analysis was performed using Affymetrix Expression Console™ software that supports probe set summarization and CHP file generation of 3^′^ expression using the MAS5 Statistical algorithm. Affymetrix microarrays contain the hybridization, labeling and housekeeping controls that help determine the success of the hybridizations. The Affymetrix Expression Analysis algorithm uses the Tukey’s biweight estimator to provide a robust mean Signal value and the Wilcoxon’s rank test to calculate a significance or p-value and Detection call for each probe set. The Detection p-value is calculated using a Discrimination Score [R] for all probes. The Discrimination Score is a basic property of a probe pair that describes its ability to detect its intended target. It measures the target-specific intensity differences of the probe pair (perfect match (PM) – mismatch (MM)) relative to its overall hybridization intensity (PM + MM). Background estimation is provided by a weighted average of the lowest 2% of the feature intensities. Mismatch probes are utilized to adjust the perfect match (PM) intensity. Linear scaling of the feature level intensity values, using the trimmed mean, is the default to make the means equal for all arrays being analyzed. False-negative and false-positive rates are minimized by subtracting nonspecific signal from the PM probe intensities and performing an intensity-dependent normalization at the probe set level. Three chips were used for each experimental group: ipsilateral, contralateral and naïve control. The datasets produced by the Affymetrix software contain gene identifiers and corresponding expression values. The datasets used for this study can be accessed in the Gene Expression Omnibus (http://www.ncbi.nlm.nih.gov/geo/) of the National Center for Biotechnology Information (NCBI) with accession number GSE45997. These data were analyzed in Microsoft Excel for calculation of fold change and whether the genes were confirmed as present in the tissue sample (as determined by the Affymetrix software). Genes in the injured brain that increased or decreased in expression by 2-fold or more compared to controls and were present in either all 3 ipsilateral samples or all 3 contralateral samples were identified. The gene datasets that were generated were ipsilateral vs naïve (TBI-I) and contralateral vs naïve (TBI-C) fold changes.

### Principal component analysis

PCA was carried out using the Gene Expression Similarity Investigation Suite software (Genesis; Graz University of Technology). The microarray datasets were analyzed and a matrix was constructed to determine distribution of variants. To compute the principal components (PCs), Genesis calculates the n eigenvalues and their corresponding eigenvectors are calculated from the (n x n) distance matrix using Singular Value Decomposition (SVD), where n = number of genes [40]. The score for each experimental dataset per PC was determined by multiplying the initial expression value by the Genesis calculated eigenvector for each gene and adding up the resulting values. The scores were plotted on the X, Y, and Z axes resulting in a 3D scores plot where the coordinates are (PC1 score, PC2 score, PC3 score).

### Ingenuity pathway analysis

The gene datasets were analyzed using Ingenuity Pathway Analysis (Ingenuity^®^ Systems, http://www.ingenuity.com) and overlaid onto a global molecular network developed from information contained in the Ingenuity Knowledge Base. Fischer’s exact test was used to calculate a p-value determining the probability that each biological function and/or disease assigned to that network is due to chance alone. The functions, canonical pathways, and gene networks that were most significant to the dataset were identified. Gene expression profiles were overlaid on the canonical pathway and gene network figures to reveal similarities and dissimilarities in their gene expression patterns. Gene networks were also created using Ingenuity Knowledge Base to further understand specific interactions between our genes of interest.

### TBI-I/TBI-C ratio

We used the following formulas to calculate the ratio of TBI-I to TBI-C fold changes: (1) Gene increased on both sides: ratio = (TBI-I)/(TBI-C); (2) Gene decreased on both sides: ratio = 1/[(TBI-I)/(TBI-C)]; and (3) Gene increased ipsilaterally and decreased contralaterally: ratio = (TBI-I)/-[1/(TBI-C)].

### Histology and immunohistochemistry

At 24 h post injury, rats were anesthetized with an intraperitoneal injection of a ketamine:xylazine:acetylpromazine cocktail (50:10:1.67 mg/kg respectively) and perfused transcardially with saline followed by cold 4% paraformaldehyde solution in PBS for 30 min. Brains were quickly removed and cryoprotected in 30% sucrose. The brains were then frozen in OCT mounting medium and stored until sectioning. Coronal sections of 20 μm thickness were cryosectioned from the perilesional brain area of each animal. Sections were mounted on slides which were stored at -80°C until further processed. Fluoro-Jade^®^ B (FJB; AG310, Millipore, Billerica, MA) labeling was performed as previously described [20]. Immunohistochemical localization of macrophages and activated microglia was performed using antibodies against ED-1 (1:500, MAB1435, Millipore) and CD11b (1:500; CBL1512, Millipore). After rinsing in 0.01 M PBS, sections were blocked with buffer containing 5% normal goat serum and 0.3% triton-x 100 for 1 h at 4°C and then incubated for 1 h at 37°C with the primary antibodies. Sections incubated with antibodies to ED-1 and CD11b were washed with PBS and incubated with DyLight 594 and DyLight 488 conjugated goat anti-mouse secondary antibodies, respectively (1:400; Jackson ImmunoResearch Laboratory, West Grove, PA) for 1 h at room temperature. Negative control sections for immunohistochemistry were incubated with the secondary antibody only (no primary antibodies). A Zeiss microscope equipped with a CCD camera (Carl Zeiss Microimaging Inc, Thornwood, NY) was used to capture digital images of the sections.

## Abbreviations

CCI: Controlled cortical impact; FJB: Fluoro-Jade B; GIH: Gene interaction hierarchy; GOI: Genes of interest; IPA: Ingenuity Pathway Analysis; IR: Inflammatory response; PCA: Principal component analysis; TBI: Traumatic brain injury; TBI-C: Contralateral vs naïve gene dataset; TBI-I: Ipsilateral vs naïve gene dataset.

## Competing interests

The authors declare that they have no competing interests.

## Authors’ contributions

TEW, GDF and BDF were responsible for overall study design and execution. TEW, MCS and MCL were responsible for the animal models, sample preparation and histological analysis. TEW, GDF, ASG and BDF were responsible for carrying out microarray studies and bioinformatic data analysis. The manuscript was written by TEW and BDF. All authors have read and approved the final manuscript.

## Supplementary Material

Additional file 1 **GOI (genes of interest) network created in IPA. **This is the resulting network when IPA connected our 114 GOI using only direct (1st order) connections between the genes. 95 of the GOI formed an interconnected network, leaving 19 “orphan” genes. Click here for file

Additional file 2 **Primary tier example for how direct connections were counted. **This figure shows an example of how we calculated the number of direct connections for a gene in our GOI network. In IPA, the gene in question was selected (JAK2 in this example). Then, its direct connections were selected by right clicking on JAK2 and using the "select nearest neighbors" option (highlighted in blue). A list of the selected genes was exported and JAK2 was removed from the list (upper right corner). The remaining genes were counted (15 in this example) and JAK2 was ranked in the gene interaction hierarchy (primary tier) by this number. Click here for file

Additional file 3 **Secondary tier example for how direct connections were counted. **This figure shows an example of how we calculated the number of direct connections for a gene in our GOI network. In IPA, the gene in question was selected (IRF2 in this example). Then, its direct connections were selected by right clicking on IRF2 and using the "select nearest neighbors" option (highlighted in blue). A list of the selected genes was exported and IRF2 was removed from the list (upper right corner). The remaining genes were counted (7 in this example) and IRF2 was ranked in the gene interaction hierarchy (secondary tier) by this number. Click here for file
