# Supplementary figures and images for: Gene expression patterns following unilateral traumatic brain injury reveals a local pro-inflammatory and remote anti-inflammatory response
Source: BMC Genomics. 2013 Apr 25;14:282. doi: 10.1186/1471-2164-14-282 (PMC3669032; doi:10.1186/1471-2164-14-282)

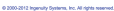

Supplement: Additional file 2 — Primary tier example for how direct connections were counted. This figure shows an example of how we calculated the number of direct connections for a gene in our GOI network. In IPA, the gene in question was selected (JAK2 in this example). Then, its direct connections were selected by right clicking on JAK2 and using the "select nearest neighbors" option (highlighted in blue). A list of the selected genes was exported and JAK2 was removed from the list (upper right corner). The remaining genes were counted (15 in this example) and JAK2 was ranked in the gene interaction hierarchy (primary tier) by this number. [file 1471-2164-14-282-S2.pdf]

**Direct Connections  
for IRF2**

CDKN1A

ERAP1

IL1B

IRF1

ISG15

PSMB8

PSMB9

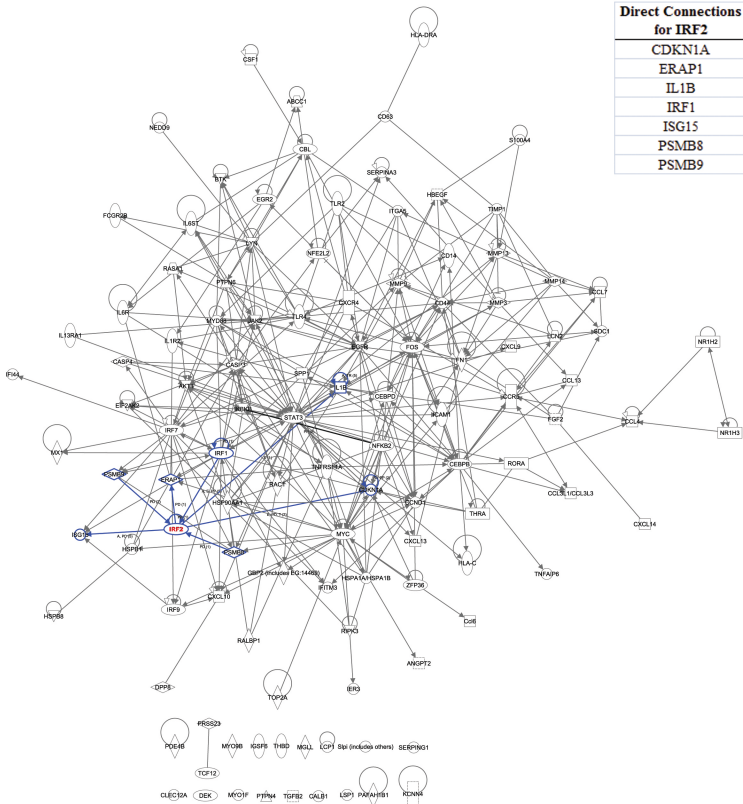

Supplement: Additional file 3 — Secondary tier example for how direct connections were counted. This figure shows an example of how we calculated the number of direct connections for a gene in our GOI network. In IPA, the gene in question was selected (IRF2 in this example). Then, its direct connections were selected by right clicking on IRF2 and using the "select nearest neighbors" option (highlighted in blue). A list of the selected genes was exported and IRF2 was removed from the list (upper right corner). The remaining genes were counted (7 in this example) and IRF2 was ranked in the gene interaction hierarchy (secondary tier) by this number. [file 1471-2164-14-282-S3.pdf]
