# Supplementary material for: Gene expression patterns following unilateral traumatic brain injury reveals a local pro-inflammatory and remote anti-inflammatory response
Source: BMC Genomics. 2013 Apr 25;14:282. doi: 10.1186/1471-2164-14-282 (PMC3669032; doi:10.1186/1471-2164-14-282)
Supplement: Additional file 1 — GOI (genes of interest) network created in IPA. This is the resulting network when IPA connected our 114 GOI using only direct (1st order) connections between the genes. 95 of the GOI formed an interconnected network, leaving 19 “orphan” genes. [file 1471-2164-14-282-S1.pdf]

IR GOI Network

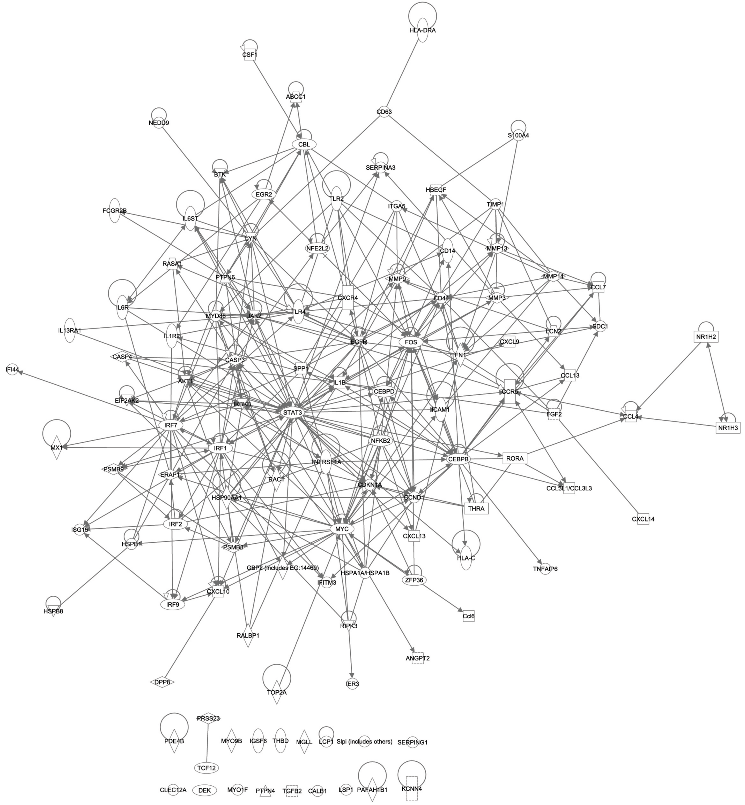

© 2000-2012 Ingenuity Systems, Inc. All rights reserved.
